# Butyrate limits inflammatory macrophage niche in NASH

**DOI:** 10.1038/s41419-023-05853-6

**Published:** 2023-05-18

**Authors:** Ankita Sarkar, Priya Mitra, Abhishake Lahiri, Tanusree Das, Jit Sarkar, Sandip Paul, Partha Chakrabarti

**Affiliations:** 1 Division of Cell Biology and Physiology, CSIR-Indian Institute of Chemical Biology, Kolkata, India; 2 Division of Structural Biology & Bioinformatics, CSIR-Indian Institute of Chemical Biology, Kolkata, India; 3 Elucidata, New Delhi, Delhi 110017 India; 4 JIS Institute of Advanced Studies & Research, Kolkata, India; 5 Present Address: Academy of Scientific and Innovative Research (AcSIR), Ghaziabad, 201002 India

**Keywords:** Non-alcoholic steatohepatitis, Chronic inflammation

## Abstract

Immune cell infiltrations with lobular inflammation in the background of steatosis and deregulated gut-liver axis are the cardinal features of non-alcoholic steatohepatitis (NASH). An array of gut microbiota-derived metabolites including short-chain fatty acids (SCFA) multifariously modulates NASH pathogenesis. However, the molecular basis for the favorable impact of sodium butyrate (NaBu), a gut microbiota-derived SCFA, on the immunometabolic homeostasis in NASH remains elusive. We show that NaBu imparts a robust anti-inflammatory effect in lipopolysaccharide (LPS) stimulated or classically activated M1 polarized macrophages and in the diet-induced murine NASH model. Moreover, it impedes monocyte-derived inflammatory macrophage recruitment in liver parenchyma and induces apoptosis of proinflammatory liver macrophages (LM) in NASH livers. Mechanistically, by histone deactylase (HDAC) inhibition NaBu enhanced acetylation of canonical NF-κB subunit p65 along with its differential recruitment to the proinflammatory gene promoters independent of its nuclear translocation. NaBu-treated macrophages thus exhibit transcriptomic signatures that corroborate with a M2-like prohealing phenotype. NaBu quelled LPS-mediated catabolism and phagocytosis of macrophages, exhibited a differential secretome which consequently resulted in skewing toward prohealing phenotype and induced death of proinflammatory macrophages to abrogate metaflammation in vitro and in vivo. Thus NaBu could be a potential therapeutic as well as preventive agent in mitigating NASH.

## Introduction

Non-alcoholic fatty liver disease (NAFLD) is a continuum of several stages of chronic liver anomalies starting from simple steatosis, progressing toward NASH and ultimately leading toward end-stage fibrotic liver disease, cirrhosis, and hepatocellular carcinoma [1]. Attribution of inflammation leads the progression of simple steatosis toward aggressive and potentially irreversible NASH stage with cardinal features of hepatocellular death, profuse immune cell infiltration, and perisinusoidal fibrosis. Among the gamut of pathogenetic events leading to NASH, maladaptation of gut-liver axis including increased gut permeability and gut microbial dysbiosis has recently emerged as a critical patho-etiological factor [2]. How does altered metabolism in gut microbial ecology interacting with host cell and perturbing hepatic immunometabolic homeostasis and metaflammation is, however, still elusive [3]. Despite the advancement in understanding the mechanistic perspective of disease pathogenesis, due to highly variable disease progression rate and varied clinical manifestations, sketchy knowledge is available for the development of effective treatment strategies [4].

In pathogenesis of NASH, the liver resident tolerogenic Kupffer cells play pivotal role by facilitating homing of monocytes via chemokine signaling and aid in harboring heterogeneous macrophage population [5]. Depending on the microenvironment, macrophages polarize toward different phenotype and exert different functional repertoire like classically activated M1 like, alternatively activated M2 like, regulatory or prohealing type. These functional diversities are highly dynamic because of inherent plasticity of macrophage population [6] and the spatiotemporal predominance of one particular macrophage phenotypic depends on the stage of NAFLD progression [7].

SCFAs are known to alleviate the MCD diet-induced NASH pathogenesis by AMPK activation mediated induction of fatty acid oxidation gene cascade [8]. NaBu is a bona fide gut microbiota-derived SCFA majorly metabolized by colonocytes as principal energy source and has shown anti-inflammatory effect on human monocyte and on LPS-activated endothelial cells involving GPR41/43 activation by inhibiting HDAC [9–11]. By reciprocally targeting HDAC2 in osteoclasts and HDAC8 in T cells, NaBu has been shown to ameliorate rheumatoid inflammation. It also resulted in enhanced iTreg generation by augmenting histone acetylation and activity of CPT1A [12, 13]. NaBu protected high-fat diet-induced NASH by upregulating hepatic GLP-1R expression and attenuation of inflammation by improving gut barrier function along with reduction in circulating endotoxin levels [14, 15]. Moreover, by targeting noncanonical TGF-β pathway in human HSC, NaBu showed antifibrotic property and promotes antimicrobial host defense program of macrophage to maintain the intestinal homeostasis [16, 17]. Oral supplementation of NaBu also protects mice from western-style diet-induced NASH pathogenesis [18]. However, direct effects of NaBu in LM and its impact on the NASH-mediated metaflammation is not known. Here we seek to decipher the molecular basis of anti-inflammatory response of NaBu on NASH pathogenesis and examine its potential therapeutic benefits.

## Results

### NaBu suppresses macrophage driven inflammation in NASH

**Fig. 1:**
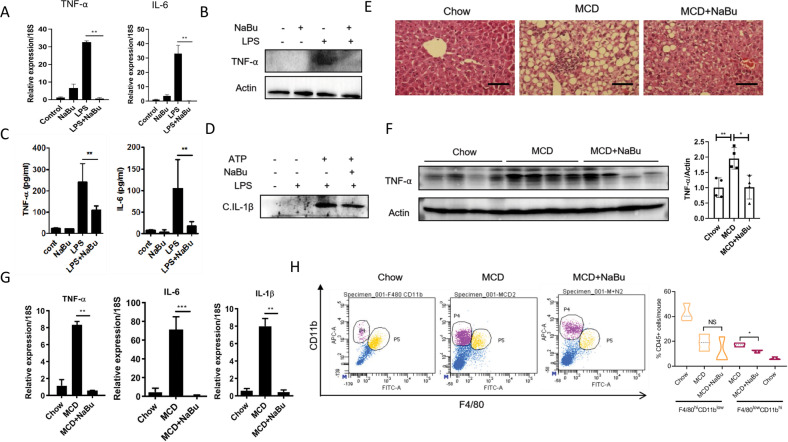
NaBu attenuates LM activation in NASH. **A**–**C** Isolated murine LMs were pretreated with NaBu (3 mM) for 30 min followed by LPS (1 μg/ml) stimulation for 4 h. Gene expressions were determined by qPCR and normalized to 18S (**A**); Expression of TNF-α was assessed by immunoblotting (**B**); Culture supernatants were analyzed for the presence of TNF-α and IL-6 by ELISA (**C**). **D** Inflammasome activation was induced in isolated LMs with dual stimulation of LPS (1 μg/ml) for 4 h followed by ATP (5 mM) for 30 min and supernatants were analyzed for the presence of cleaved IL-1β by immunoblotting. **E**–**G** Mice were fed with MCD diet for 2 weeks with simultaneous oral gavage of NaBu (100 mg/kg body weight) [*n* = 6 for each group]. Staining of liver sections with hematoxylin and eosin (scale bars: 200 μm) of chow fed control (left), MCD fed (middle), and MCD+NaBu group (right) of mice (**E**). TNF-α expression in liver lysates of treatment groups analyzed by immunoblotting (left) and relative band intensities of TNF-α (right) (**F**). qPCR for gene expression (**G**). **H** Flow cytometric analysis of isolated non-parenchymal fraction from mice liver gated for CD45+ cells. Two distinct populations identified as F4/80^high^ CD11b^low^ (P5) and F4/80^low^ CD11b^high^ (P4) as resident and recruited population of macrophages, respectively. Values were presented as mean ± SD, *P < 0.05; **P < 0.01; NS, not significant.

To assess the immunomodulatory effect of NaBu in context of liver pathophysiology isolated murine LM were treated with NaBu and followed by LPS stimulation. NaBu markedly suppressed LPS-induced proinflammatory gene expression (TNF-α, IL-6) as well as secretion of these cytokines (Fig. 1A, C). TNF-α protein expression was also diminished under the same experimental condition (Fig. 1B). As inflammasome activation represents an important axis in maintaining immune homeostasis, dual stimulation was given to the LM with LPS in tandem with ATP to induce inflammasome and the secreted cleaved IL-1β levels was determined in culture supernatant via immunoblotting. Similar to inflammatory gene expressions, NaBu also reduced the inflammasome activation (Fig. 1D). For further exploration of anti-inflammatory property of NaBu in vivo, we developed MCD diet-fed mouse NASH model gavaged with NaBu (Fig. 1E). In agreement with in vitro findings, NaBu significantly reduced hepatic TNF-α protein expression (Fig. 1F) and proinflammatory gene expressions (Fig. 1G) along with concomitant reduction in steatosis and infiltrations of inflammatory cells in the liver parenchyma (Fig. 1E). To assess whether NaBu differentially modulates hepatic recruited macrophage population that predominantly drive the inflammatory insult in NASH, gated CD45+ cells were flow cytometrically analyzed by F4/80 and CD11b antibodies, the standard readouts for LM [19, 20]. While LMs from MCD diet group showed two distinct populations, F4/80^high^CD11b^low^ and F4/80^low^CD11b^high^, representing resident and recruited LMs, respectively, a significantly reduced recruited macrophage homing occurred in NaBu gavaged group (Fig. 1H).

**Fig. 2:**
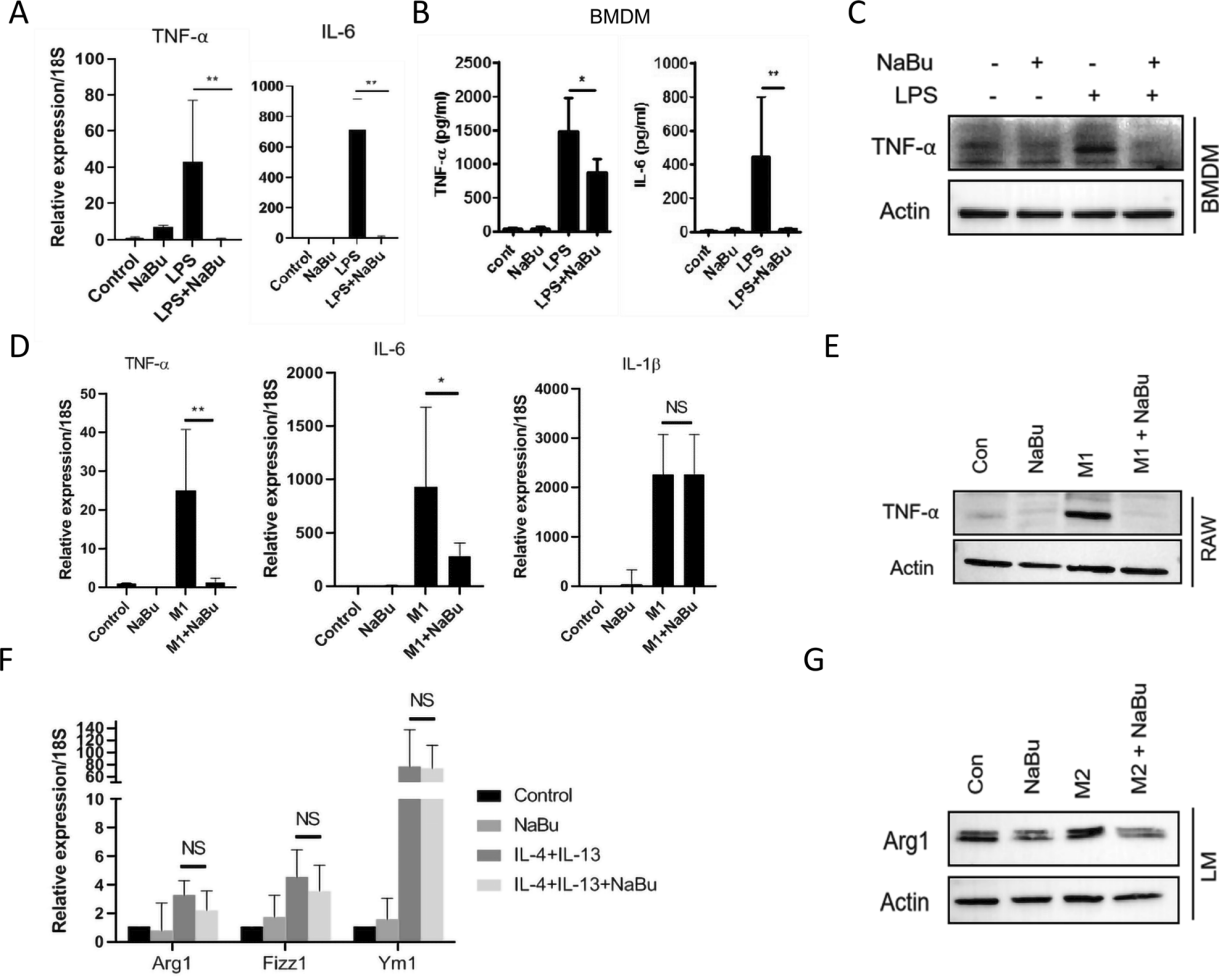
NaBu suppresses LPS-induced macrophage activation and polarization. **A**–**C** Isolated murine BMDMs were pretreated with NaBu (3 mM) for 30 min followed by LPS (1 μg/ml) stimulation for 4 h. Gene expressions were normalized to 18S (**A**). Supernatants of aforementioned treatment conditions were analyzed for the presence of TNF-α and IL-6 by ELISA (**B**). Expression of TNF-α was assessed by immunoblotting (**C**). **D**, **E** Gene expressions in NaBu pretreated M1 polarized (LPS, 1 μg/ml; IFN-γ, 50 ng/ml) BMDM (**D**) and TNF-α protein levels by immunoblotting RAW264.7 (**E**). **F** qPCR analysis for mentioned genes in IL-4 and IL-13 stimulated BMDM and gene expression was normalized to 18S. **G** Immunoblot of TNF-α in M2 polarized LM. Values were presented as mean ± SD, *P < 0.05; **P < 0.01; NS, not significant.

We further appraised whether the anti-inflammatory effects of NaBu is exclusive for LMs or a generic phenomenon relevant to other types of macrophages. Similar to LM, NaBu elicited a robust anti-inflammatory effect on the LPS-challenged BMDM by suppressing TNF-α and IL-6 expression and secretion (Fig. 2A–C). Consistently, NaBu dose-dependently suppressed cytokine expression and secretion RAW264.7 cells (Supplementary Figure 1A–C). Next, we examined the impact of NaBu on the classically or alternatively activated macrophage. NaBu potently suppressed the proinflammatory cytokine expression and secretion from the classically activated M1 BMDM and RAW264.7 cells (Fig. 2D, E). Interestingly, NaBu did not alter the expression of IL-1β in M1 skewed macrophages (Fig. 2D). Conversely, NaBu did not show any significant change in the IL-4 and IL-13 stimulated alternatively activated M2 signature genes, viz. Arg1, Fizz1 and Yim1and Arg1 protein expressions (Fig. 2F, G). Taken together, NaBu imparts profound anti-inflammatory effects in LPS or classically activated macrophage in vitro and in NASH.

### NaBu modulates acetylation of p65 and its chromatin recruitment

**Fig. 3:**
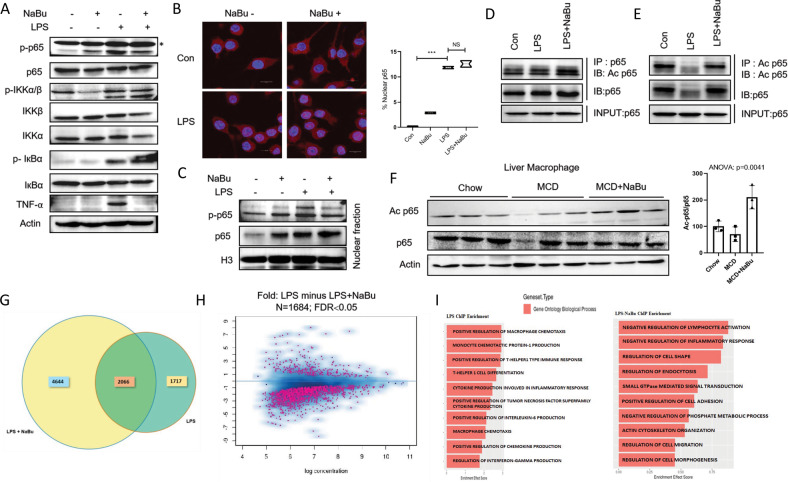
NaBu augments p65 acetylation and its chromatin recruitment. **A** RAW264.7 cells were pretreated with NaBu (3 mM) for 30 min followed LPS (1 μg/ml) stimulation for 4 h and canonical NF-κB pathway was analyzed by immunoblotting via for the expressions of phospho-p65, p65, p-IKKα/β, IKKβ, IKKα, p-IκBα, IκBα. **B** Nuclear localization of p65 was assessed by immunofluorescence staining of p65 in LPS-stimulated RAW264.7 cells with and without pretreated NaBu and images were taken by using confocal microscopy (scale bars, 10 μm). **C** LPS stimulated RAW264.7 cells pretreated with NaBu for 30 min, p65 and phospho-p65 expressions were analyzed by immunoblotting in fractionated nuclear extracts. **D**, **E** Expression of acetylated p65 in control, LPS, and LPS+NaBu RAW264.7 cells by immunoblotting (IB, immunoblot; IP, immunoprecipitation). **F** Expression levels of acetylated p65 in isolated non-parenchymal fraction of liver from Chow fed, MCD fed, and MCD+NaBu fed mice (left). Relative band intensities of acetylated p65 (*n* = 3, right). **G** Venn diagram showing overlapped promoter recruitment site of p65 between LPS and LPS+NaBu treated ChIPseq data. **H** Volcano plot showing the p65 promoter recruitment status of LPS and LPS+NaBu treated RAW264.7 cells (FDR < 0.05). **I** Representation of ChIP enrichment analysis and the upregulated pathways with gene sets of Gene Ontology Biological Process of LPS and LPS+NaBu treated RAW264.7 cells.

To unveil the molecular basis of effusive anti-inflammatory property of NaBu, we first blocked the two putative G-protein-coupled NaBu receptors GPR41 and GPR43, respectively, by SHB and GLPG [21]. Although NaBu treatment induced GPR41 and GPR43 gene expressions, levels of them did not change following LPS stimulation (Supplementary Figure 2A). Treatment of antagonists either alone or in combination failed to augment NaBu-mediated suppression of cytokine expressions under LPS stimulation (Supplementary Figure 2B, C). We next examined whether NaBu modulates the LPS-mediated activation of canonical NF-κB pathway in inducing proinflammatory axis in macrophages [22]. LPS stimulation initiates a cascade of phosphorylation events of IKK and IκB leading to nuclear translocation of transcription factor p65 which consequently drives proinflammatory gene expression program. NaBu treatment did not alter LPS induced either phosphorylation of IKK, IκB, and p65 or latter’s nuclear localization despite having remarkable drop in TNF-α expression (Fig. 3A–C). These results indicate that anti-inflammatory response of NaBu is independent of both cell surface receptor and canonical NF-κB pathways.

NaBu is a known histone deacetylase (HDAC) inhibitor with somewhat specific toward HDAC3 and that in turn can regulate acetylation of p65 [23, 24]. Seven lysine residues are available for p65 acetylation and among them lysine 310 controls its transcriptional activity [25]. We first confirmed that ectopic expression of both HDAC1 and HDAC3 could diminish acetylation of p65 in HEK293T cells (Supplementary Figure 3A). Next, we treated LPS-stimulated macrophages with a specific HDAC3 inhibitor RGFP966. Consistent with NaBu treatment, RGFP966 dose-dependently suppressed LPS induced TNF-α expression (Supplementary Figure 3B) as well as abrogated proinflammatory gene expression in LPS stimulated RAW264.7 in the background of siRNA mediated HDAC3 silencing (Supplementary Figure 3C), a phenomenon syncing with the earlier observation that HDAC3 null macrophages engendered alternatively activated macrophage phenotype [26]. Immunoprecipitation of p65 or acetylated p65 showed marked reduction of lysine 310 acetylation upon LPS stimulation while pretreatment with NaBu had restored rather augmented cellular acetylated p65 levels (Fig. 3D, E). Consistent with in vitro data, we observed significantly enhanced acetylation of p65 in LMs isolated from NaBu-treated mice fed with MCD diet for 2 weeks (Fig. 3F). To further assess whether acetylation of p65 is responsible for NaBu-mediated transcriptional rewiring in macrophages, we performed ChIP of LPS stimulated cells pretreated with NaBu. LPS-dependent p65 recruitment to TNF-α and IL-6 promoters were abrogated by NaBu treatment which, however, remain unaltered for IL-1β promoter engagement (Supplementary Figure 3D). To further identify the genome-wide p65 promoter occupancy, we performed ChIP-seq. Interestingly p65 was recruited to a greater number of gene promoters in NaBu pretreated cells compared to LPS stimulated cells (1402 promoters in NaBu pretreated condition and 282 promoters in LPS stimulated cells; Fig. 3G, H). Based on ChIP enrichment score, we next performed gene set enrichment analysis (GSEA). Expectedly, in LPS-stimulated macrophages the significantly upregulated pathways (with positive Enrichment effect score) are akin to the classical proinflammatory macrophage activation such as Positive regulation of Tumor Necrosis Factor Superfamily Cytokine Production, Positive regulation of Chemokine production, Macrophage Chemotaxis. In contrast, proinflammatory pathways were absent and instead a different set of pathways such as Regulation of Cell Shape, Actin Cytoskeleton Organization, Negative Regulation of Inflammatory Response emerged in NaBu-treated macrophages (Fig. 3I). Our analysis thus indicates that NaBu suppresses LPS-induced proinflammatory promoter occupancy of p65 through its disparate chromatin recruitment. Taken together, our results reveal that by inhibiting HDAC3, NaBu modulates p65 acetylation and its differential promoter occupancy.

### NaBu instills a prohealing transcriptional program in macrophages

**Fig. 4:**
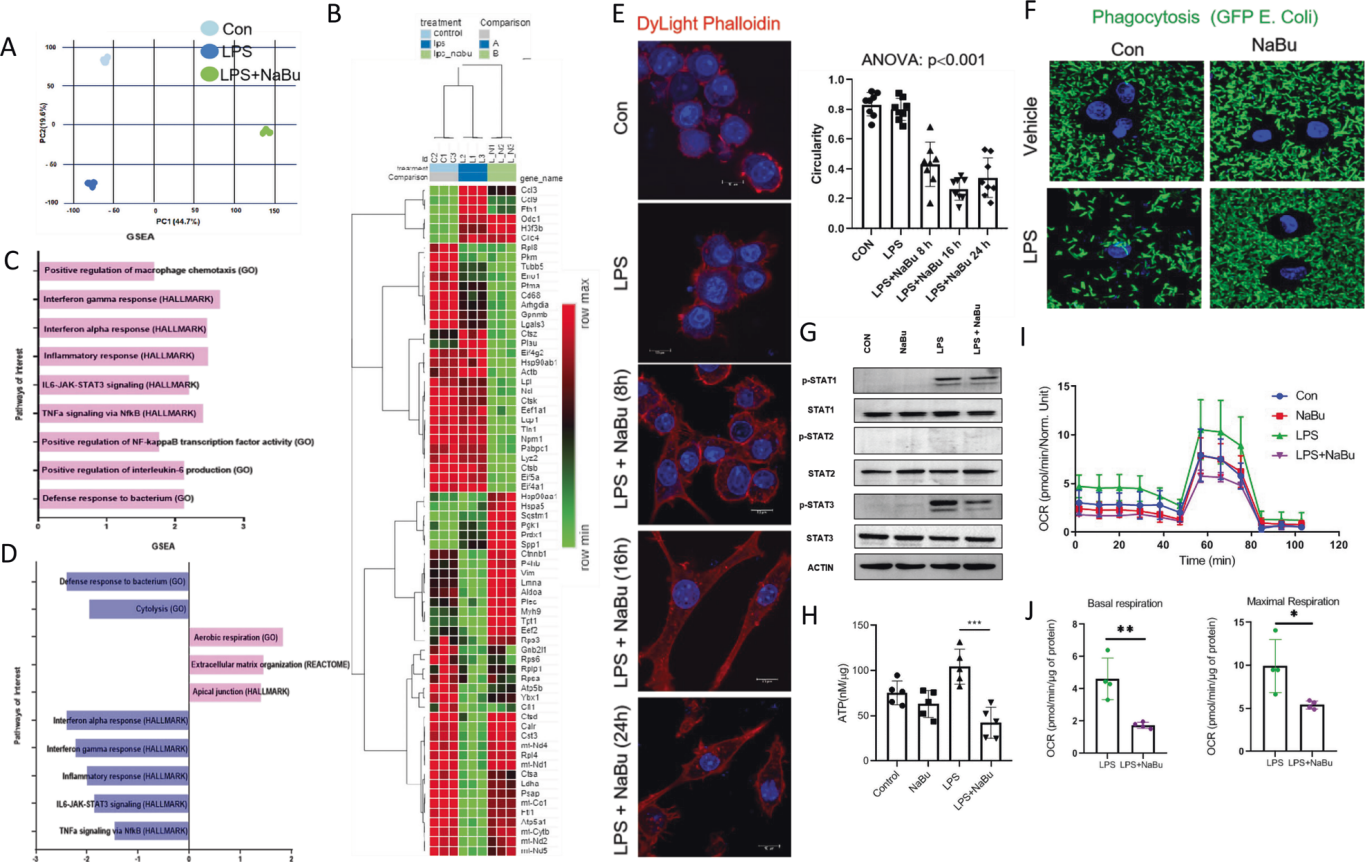
NaBu rewires transcriptional landscape and skews macrophages toward prohealing phenotype. **A** PCA analysis of the RNA seq data of control, LPS, and LPS+NaBu treated RAW264.7 cells. **B** Heat map of the differentially expressed genes identified from RNA seq data of control, LPS, and LPS+NaBu treated cells. **C** GSEA of different pathway databases of Control vs. LPS RNA seq data. Pink bars indicating upregulated pathways. **D** GSEA of different pathway databases of LPS vs. LPS+NaBu, pink bars represented upregulated and blue bars represented the downregulated pathways. **E** DyLight Phalloidin staining of NaBu pretreated, LPS-activated RAW264.7 cells for various time points (8, 16, 24 h) [Scale bars, 10 μm]. Quantification of cellular circularity (right). **F** Phagocytosis assay, GFP-tagged *E. coli* were incubated with NaBu pretreated LPS stimulated RAW264.7 cells for 3 h followed by images were taken by confocal microscopy [Scale bars, 10 μm]. **G** Expressions of STAT1, p-STAT1, STAT2, p-STAT2, STAT3, and p-STAT3 in NaBu treated (3 mM) followed by LPS-activated RAW264.7 cells. **H** ATP levels determined in NaBu pretreated LPS stimulated RAW264.7 cells. **I**, **J** Oxygen consumption rate (OCR) measured under basal and maximal state (FCCP, 2.5 μg) and normalized with total protein concentration. Values were presented as mean ± SD, *P < 0.05; **P < 0.01; ***P < 0.001.

Our ChIP-seq data is suggestive of NaBu-dependent p65-driven transcriptional rewiring of macrophage activation. Toward further elucidation of NaBu-induced macrophage activation, we performed transcriptomics profiling of untreated, LPS and LPS with NaBu-treated cells. Principal Component Analysis (PCA) revealed three distinct clusters (Fig. 4A) which is consistent with the heat map of top70 differentially expressed genes showing contrasting pattern among the three groups (Fig. 4B). GSEA further revealed that LPS stimulation significantly upregulated a number of proinflammatory pathways such as TNF-α signaling via NF-κB, IL-6 JAK-STAT3 Signaling, Inflammatory response (Fig. 4C) most of which were correspondingly downregulated upon NaBu pretreatment (Fig. 4D), indicating reversal of classical macrophage activation and possible emergence of a unique macrophage phenotype. LPS stimulation leads to the induction of proinflammatory gene cascade and among that repertoire, IL-6 interacts with heterodimeric receptor followed by JAK-mediated STAT3 phosphorylation to further potentiate phospho-STAT3 dependent proinflammatory gene expression [27]. We have pretreated the RAW264.7 cells with NaBu followed by LPS stimulation and checked the phosphorylation status of STAT3 and observed reduced phosphorylated STAT3 in NaBu pretreated condition which is corroborating with our transcriptomics GSEA data while phosphorylation of STAT1 and STAT2 remained unaltered (Fig. 4I).

For the phenotypic and functional characterization of NaBu-induced macrophage, we next assessed the cellular cytoskeleton using phalloidin which specifically binds to F-actin. While LPS stimulation led to activated spherical shape of macrophages, treatment of NaBu induced a temporally perceptible elongated cellular architecture (Fig. 4E). Our results are thus consistent with published data that structural alternations of macrophages synergize with phenotypic switching and particularly elongated structure is an indicative of anti-inflammatory rather prohealing (M2 like) type of remodeling [28]. Classically activated or M1 macrophages have enhanced phagocytic activity to wall off the inflammatory insults and that could also differentiate the phenotypic class of macrophages [29]. We next stimulated the cells with LPS and incubated with GFP-*E. coli* to microscopically access the state of phagocytosis. LPS-stimulated cells expectedly showed enhanced whereas NaBu pretreated cells following LPS stimulation displayed markedly reduced phagocytosis of GFP-*E. coli* (Fig. 4F), further indicating emergence of prohealing type of class switching. As metabolic activity of macrophages dictates their phenotype and functional outcome [30], we next measured cellular respiration, lactate secretion, and ATP levels in NaBu pretreated cells upon LPS stimulation. There was a significant drop in ATP concentration (Fig. 4G), lactate production (Supplementary Figure 4A) as well as markedly reduced basal and maximal respiration in NaBu-treated cells (Fig. 4H, J), indicative of compromised oxidative phosphorylation. However, we did not find any change in either in mitochondrial membrane potential or in expression levels of respiratory complexes (Supplementary Figure 4B, C). Collectively, NaBu imparts a transcriptional program that skews LPS-induced proinflammatory macrophages toward a prohealing phenotype.

### NaBu induces apoptosis of proinflammatory macrophages

**Fig. 5:**
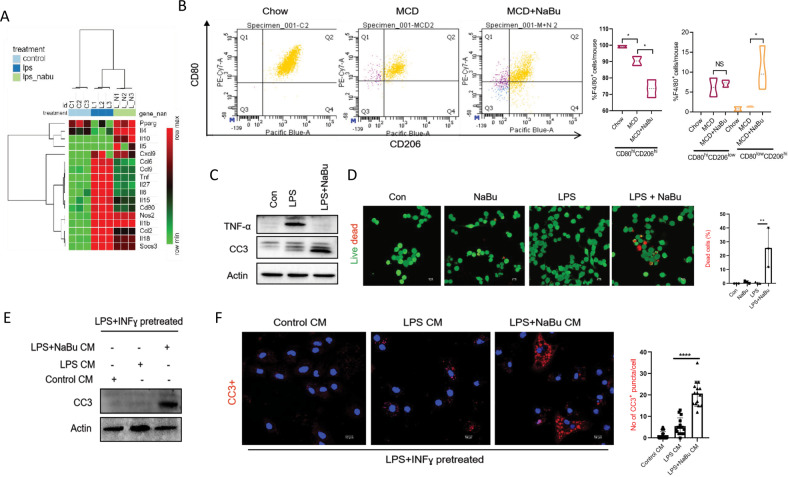
NaBu induces apoptosis of proinflammatory macrophages. **A** Heat map of selected M1- and M2-like genes from RNA seq data. **B** Flow cytometric analysis of isolated non-parenchymal fraction from Chow fed, MCD fed, NaBu gavaged MCD fed mice liver and gated for all F4/80+ cells followed by CD80^hi^CD206^low^ (M1 like) and CD80^low^CD206^hi^ (M2 like) populations (left); Statistical summary of flow cytometry data (right, *n* = 4). **C** Expression of TNF-α and cleaved caspase 3 by immunoblotting pretreated with NaBu followed by LPS stimulation with total incubation of 8 h in RAW264.7 cells. **D** Apoptosis was assessed by Live/Dead assay in RAW264.7 cells pretreated with NaBu followed by LPS stimulation (left). Quantification of % of dead cells (right). **E**, **F** Expression of CC3 by immunoblotting (**E**) and immunofluorescence microscopy (**F**) in LMs stimulated with LPS (1 μg/ml) and IFN-γ (50 ng/ml) for 16 h followed by treatment with conditioned media of control, LPS, and LPS+NaBu treated LMs for 10 h. Values were presented as mean ± SD, *P < 0.05; **P < 0.01; ***P < 0.001; ****P < 0.0001; NS, not significant.

To further examine the prohealing phenotype, we analyzed transcriptomic datasets for the expressions of signature genes for M1 and M2 macrophages. NaBu diminished expressions of a subset of proinflammatory genes such as *tnf, il6, Cd80* and enhanced expressions of anti-inflammatory signature genes such as *pparg*, *il4*, *il10* (Fig. 5A). Consistent with ChIP data, NaBu had no or minimal impact on the expression of *il1b*, *Nos2*, *il18*, suggesting that NaBu did not completely transform the cells to M2 phenotype. To better understand the M1-M2 paradigm in vivo, LMs were examined from MCD diet fed and concurrently NaBu gavaged animals. Following gating the F4/80+ population (macrophage marker), CD80+ (M1 marker) and CD206+ (M2 marker) cells were analyzed. LMs from NaBu gavaged animals showed an emerging population of CD206+ cells compared to LMs isolated from MCD diet-fed group without any significant difference of M1 population (Fig. 5B). We also observed a significant decline in the number of F4/80+ population from NaBu gavaged livers compared to MCD diet fed group suggesting a drop in the proinflammatory M1 like macrophages (Fig. 5B). To further assess whether NaBu promotes death of proinflammatory macrophages, we checked for the expression of cleaved caspase 3 (CC3), readout of apoptosis in the NaBu pretreated and LPS-activated RAW264.7 cells. Treatment of NaBu led to enhanced levels of CC3 with simultaneous reduction in TNF-α expression, indicating conspicuous death of LPS stimulated macrophage (Fig. 5C). Consistently, live dead assay reveled ∼25% apoptotic cells in NaBu treated cells (Fig. 5D). We next asked whether NaBu alone or the altered secretome of NaBu treated macrophage is responsible for the induction of apoptosis in the proinflammatory macrophages, LMs were polarized toward M1 cells with LPS and INF-γ and then treated with conditioned media (CM) of control, LPS, and LPS with NaBu treated LMs. CM from the LPS and NaBu co-treated cells and not CM from LPS-treated cells significantly induced CC3 protein expression as revealed by immunoblotting and immunofluorescence (Fig. 5E, F). These results were also recapitulated in RAW264.7 cells (Supplementary Figure 4D). Our findings thus suggest that secreted factors from the NaBu-treated LM are responsible for the apoptotic events which possibly represent another arm of NaBu-mediated mitigation of inflammatory insults.

### NaBu alleviates NASH by reducing the number of proinflammatory LM

**Fig. 6:**
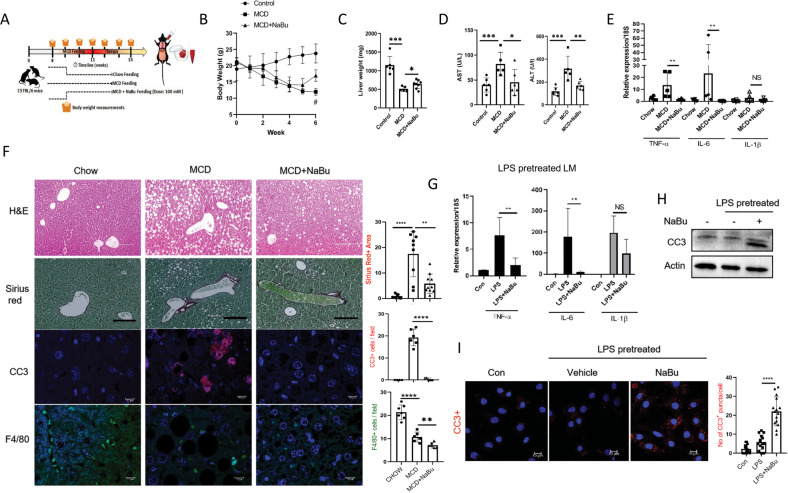
NaBu treatment reverses hallmarks of NASH. **A**–**F** Wild-type mice fed with either chow diet or with MCD diet for 3 weeks. MCD diet fed mice were randomly distributed into two groups followed by ad libitum NaBu (100 mM in drinking water) given as therapy for 3 weeks (*n* = 6/group). Schematic representation of in vivo therapeutic study (**A**). Body weight over the entire period of experimental regimen; ^#^*P* < 0.05 (**B**). Liver weight (**C**) and serum AST and ALT (**D**). qPCR analysis for mentioned genes from liver and gene expression normalized to 18S (**E**). H&E, sirius red, IF staining of cleaved caspase 3 and F4/80 of liver sections (left) (H&E, sirius red, scale bars: 200 μm); Quantifications of sirius red area, CC3+ cells and F4/80 positive cells (right) (**F**). **G**–**I** LMs stimulated with LPS for 16 h followed by NaBu treatment for 8 h. qPCR analysis for mentioned genes normalized to 18S (**G**), expression of CC3 via immunoblotting (**H**), and expression of CC3 via immunofluorescence microscopy (left), quantification of CC3+ puncta per cell (right) (**I**). Values were presented as mean ± SD, *P < 0.05; **P < 0.01; ***P < 0.001; ****P < 0.0001; ns, not significant.

NaBu potently suppressed proinflammatory gene expressions and induced apoptosis of inflammatory LMs, we thereby investigated its therapeutic potential in NASH. To this end, we fed mice with MCD diet for 3 weeks to induce NASH followed by ad libitum NaBu (100 mM in drinking water) along with MCD, for another 3 weeks (Fig. 6A). MCD diet feeding expectedly provoked persistent weight loss [31] throughout the experiment which, however, was significantly recovered upon NaBu feeding along with concomitant increase in liver weight (Fig. 6B, C). Serum AST and ALT levels were also reduced upon NaBu treatment indicating an improvement of liver injury (Fig. 6D). Liver inflammation was also mitigated as evident from gene expressions of proinflammatory markers (Fig. 6E). There were marked reductions in NASH features such as steatosis, fibrosis, and hepatocellular death (CC3+ cells) in livers of NaBu treated mice. Moreover, number of F4/80+ (macrophage marker) cells were strikingly reduced in livers of NaBu-treated animals (Fig. 6F). Number of resident macrophages usually decline upon MCD diet feeding [32] followed by augmented immune cell infiltration while NaBu treatment further reduced the numbers of F4/80+ cells. To further examine the cell autonomous therapeutic effects of NaBu, isolated LMs were first treated with LPS followed by NaBu for 8 h. NaBu treatment significantly reduced LPS-induced proinflammatory gene expressions and induced apoptosis as evidenced by CC3 levels corroborating with reduced number of F4/80+ cells in NaBu fed mice livers (Fig. 6G–I). Apoptosis of M1 Kupffer cells ensures better prognosis of alcoholic and non-alcoholic fatty liver disease [33] and that NaBu-induced death of proinflammatory LMs and resolution of inflammation further support its therapeutic potential in NASH.

## Discussion

The importance of gut-liver axis that includes gut microbial dysbiosis, altered microbial metabolites and increased gut epithelial permeability in the pathogenesis of NAFLD and NASH has now been established [1]. Among the large repertoire of microbial metabolites three SCFAs namely butyrate, propionate, and acetate have been shown to influence multiple facets of NASH pathogenesis. Although butyrate or NaBu has recently emerged as a potent agent to mitigate NASH [14], the molecular underpinning and particularly its role in the inflammatory axis of NASH pathogenesis are not known. In this study, we have unraveled the hitherto unknown molecular and cellular mechanisms and established NaBu as a potential therapeutic agent in NASH.

NaBu confers protection of LPS-induced acute liver injury and reduction of inflammatory response in intestinal macrophages and in PBMC, respectively, by involving GPR43/β arrestin-2/NF-κB axis [34] and nuclear translocation of p65 [35]. However, we did not find involvement of either the NaBu receptors (GPR41/43) or LPS-induced nuclear translocation of p65 for aforementioned robust anti-inflammatory effect putatively because of different experimental and disease contexts. Moreover, using different macrophages including LM, BMDM, and RAW264.7, we showed that mechanism of anti-inflammatory activity of NaBu is indeed generic for divergent macrophages lineage. NaBu is also a gut microbiota-derived immunomodulatory metabolite that could function as HDAC inhibitor to enhance iTreg differentiation by enhancing histone acetylation [13]. Deacetylase activity of HDAC is, however, not exclusively restricted to histone proteins but also involves deacetylation of non-histone proteins including p65. As NaBu is a known as HDAC inhibitor [13, 23] it could thereby modulate acetylation of p65 to alter cellular function. We showed enhanced acetylation rather restoration of p65 acetylation status upon NaBu treatment both in vitro and in vivo. Consistent with published data we also found that among the HDAC superfamily selective HDAC3 inhibitor RGFP966 has potent anti-inflammatory effect [36]. However, converse to NaBu, RGFP966 did not induce apoptosis of proinflammatory macrophages, indicating a HDAC3 independent death induction mechanism of proinflammatory macrophages.

NaBu treatment led to transition of macrophage phenotype in such a way that they mimic a few functional features of alternatively activated M2 macrophage with reduced phagocytosis property, modified actin cytoskeletal architecture, and compromised mitochondrial function without robust induction of anti-inflammatory gene sets. Although NaBu has been shown to facilitate M2 polarization [37], we find no or minimal impact of NaBu augmenting the IL-4 and IL-13 mediated M2 polarization of macrophage. However, we have identified emergence of M2 macrophage population in NaBu-fed livers. Together, these results indicate that anti-inflammatory effects of NaBu are at best modest in nature. In contrast, NaBu elicits a strong anti-inflammatory response to LPS stimulation and polarization to classically activated M1 macrophage. M2 polarized Kupffer cells could induce apoptosis of M1 Kupffer cells and thereby mitigates the progression of alcoholic as well as non-alcoholic fatty liver disease [33]. We observed a significantly reduced absolute number of macrophages along with a relative increase in M2 cells in NASH livers fed with NaBu suggesting that NaBu specifically induced apoptosis of proinflammatory macrophages. Moreover, the altered secretome of NaBu-treated macrophages had similar apoptosis inducing response on isolated proinflammatory LMs. Of note, following the T-helper cell phenotypes, classification of M1 and M2 macrophage populations based on certain markers in vitro does not necessarily represent the complex milieu of immunometabolic diseases including NASH [38]. We therefore surmise that NaBu skews macrophages in a way that may not recapitulate the fully polarized LM populations but toward a ‘prohealing’ phenotype that subsequently led to favorable therapeutic outcome.

**Fig. 7:**
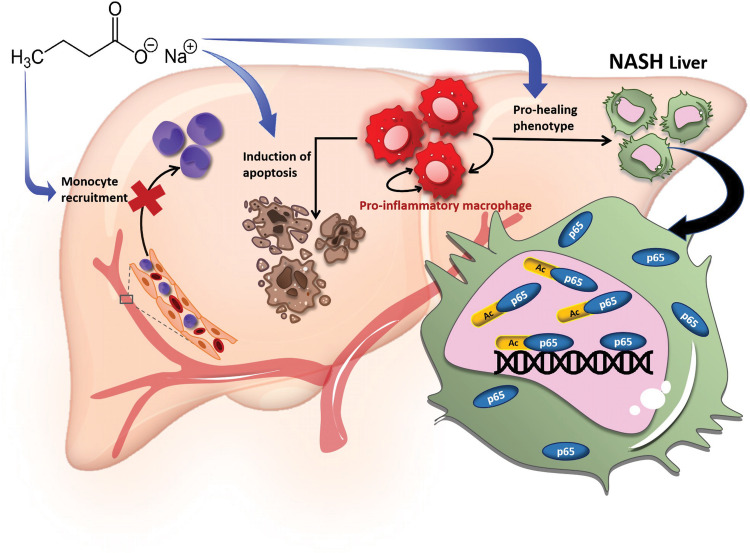
Schematic of the mechanism of NaBu in amelioration of NASH. NaBu ameliorated inflammatory insults of NASH by inhibiting proinflammatory macrophage infiltration, modulating polarization status as well as by inducing apoptosis of proinflammatory macrophages (blue arrows). The molecular mechanism of mitigated inflammatory response is hyperacetylation of p65 and its differential recruitment to proinflammatory gene promoters.

NaBu thus exhibits preventive as well as therapeutic effects in diet-induced murine NASH by mitigating inflammatory insults through curtailing proinflammatory macrophage recruitment, induction of alternatively activated M2-like macrophages and reduction of number of LMs. Notably, the anti-inflammatory activities of NaBu are attributed to at least three cellular events, viz. suppression of LPS-mediated proinflammatory gene expression, emergence of anti-inflammatory or prohealing phenotype and induction of apoptosis of proinflammatory macrophages. The latter is mediated by a reconditioned secretome of the prohealing macrophages through both autocrine and paracrine modes. At the molecular level, these cellular phenomena are mediated via augmented acetylation of transcription factor p65-dependent differential chromatin recruitment and trancriptomic rewiring (Fig. 7). Our work is also relevant in the clinical context, as fecal transplantation from lean individual to obese patients leads to the predominance of butyrate producing *Roseburia intestinalis* colonization and thereby modulating the gut microbial ecology indirectly or by direct butyrate production leading to the better clinical prognosis of NAFLD [39]. Our study thus opens up new therapeutic opportunities in NASH by targeting the gut-liver axis and exploiting the microbial metabolites.

## Materials and methods

### Animals

Protocols for animal experiments were approved by Institutional Animal Ethics Committee at CSIR-IICB under the aegis of Committee for Control and Supervision of Experiments on Animals (CPCSEA), Ministry of Environment and Forest, Government of India. Wild type 6–8-week-old C57Bl/6 male mice were housed at individually ventilated cages, with 12 h light/dark cycle at 22 ± 1 °C with ad libitum food and water. Random allocation method was used to segregate mice for different experimental groups and no blinding method was used during the experimental procedure. There was no specific exclusion criteria. To induce NASH pathogenesis, mice were fed with MCD diet for 2 weeks with simultaneous NaBu oral gavage for preventive model and for therapeutic model, 100 mM NaBu in drinking water given ad libitum for last 3 weeks of six weeks MCD diet. Blood samples were collected by cardiac puncture and serum was separated after clotting for biochemical analysis and isolated livers were cut into small pieces for downstream experiments.

### Liver enzyme assay

Serum aspartate aminotransferase (AST) and alanine aminotransferase (ALT) levels were measured using the kinetic method by commercially available kit (Randox Laboratories).

### Triglyceride estimation

Lipids were extracted from liver tissue using hexane/isopropanol (3:2, v/v) solution and triglyceride was estimated by using a kit (Randox Laboratories). Triglyceride content was normalized with respective liver weight before extraction.

### Histology, picro sirius red staining, and immunofluorescence (IF)

Paraffin-embedded liver tissue sections (thickness; 5 μm) were deparaffinised, rehydrated and processed for haematoxylin and eosin staining. For semiquantitative analysis of liver fibrosis liver sections were stained for 90 min in 0.1% (W/V) Sirius Red in saturated aqueous solution of picric acid followed by washing in acidified water. Images were taken with Evos XL Core, Invitrogen light microscope. For IF in tissue sections antigen retrieval was done by heating the slides with acidic sodium citrate buffer in microwave to aid antibody binding followed by primary antibody incubation overnight at 4 °C. Hoescht was used for counterstaining and images were taken under Leica TCS SP8 STED microscope.

### Phagocytosis assay

GFP expressing *E. coli* was induced with 100 mM IPTG for 3 h in log phase. GFP *E. coli* were supplemented at a ratio of 100 bacteria/macrophage (RAW264.7). Cells were co-incubated for 3 h at 37 °C with 5% CO_2_ to allow the bacterial uptake. Phagocytosis was stopped by adding 500 µl of ice-cold PBS followed by nuclear staining with Hoechst (Invitrogen) and viewed under Leica TCS SP8 STED microscope.

### DyLight Phalloidin staining

4% paraformaldehyde-fixed cells were treated with DyLight Phalloidin (CST, MA, USA; dilution 1:200) and 25 μg/ml Hoechst (Invitrogen) were added to the cells and incubated at 37 °C for 15 min under dark conditions. Images were taken by using Leica TCS SP8 STED microscope.

### Cell culture

LMs were isolated by in situ digestion with 0.5 mg/ml Collagenase D and resultant cell suspension was filtered through a 70 µm cell strainer and centrifuged at 250 × *g* for 5 min. The cell pellet was washed with RBC lysis buffer followed by washed in DMEM with 10% FBS, seeded onto tissue culture plates, and allowed to adhere for minimum 8 h. Non-adherent cells were removed by several washes with PBS. To isolate BMDM, dissected femur bones were kept in ice-cold PBS. A syringe filled with ice-cold PBS was used to flush out the bone marrow cells and filtered through a 70 µm nylon cell strainer to remove debris. The filtrate was centrifuged at 500 × *g* for 5 min at 4 °C, the supernatant was washed with ice-cold RBC lysis buffer and cultured in DMEM complete media with 15% of L929 cell supernatant. Cells were incubated at 37 °C with 5% CO_2_ for 3 d and were allowed to grow followed by media change with complete DMEM with 15% L929 supernatant media. Downstream experiments were done after 7 d to allow complete differentiation of BMDM. RAW264.7 (Murine peritoneal macrophage cell line purchased from ATCC) was cultured in high glucose DMEM with 10% FBS containing 1% antibiotic antimycotic solution. Media composition of the L929 and HE293A cells are same as RAW264.7 cells but for subculture purpose, 0.1% trypsin EDTA solution was used for detaching the cells. The L929 cells were used to prepare conditioned media (in FBS null media for 7 d to induce secretion of M-CSF) used for BMDM differentiation.

### Inflammasome activation

RAW264.7, LMs, and BMDM cells were primed with LPS at a concentration of 1 µg/ml for 4 h and treated with 5 mM ATP was given for 30 min as described earlier [40].

### ATP content

From cellular lysates, ATP was measured by using ATP Determination Kit (Thermo Fisher Scientific) according to the manufacturer’s protocol and was normalized with the amount of total protein. Luminescence was measured using the Synergy H1 Hybrid reader (Biotek).

### Cytokine measurement

Levels of mouse TNF-α and IL-6 were determined in culture supernatants using commercially available ELISA kits (R&D Systems), according to the manufacturer’s instructions.

### Oxygen consumption rate (OCR)

Mitostress assay was performed by washing and incubating the cells (25000 cells/ well) with XF Assay medium (supplemented with 20 mM glucose, 1 mM sodium pyruvate, and 1 mM glutamate, and without sodium bicarbonate) for 1 h. Final concentration of 2.5 mM of oligomycin, 1.5 mM of FCCP [carbonyl cyanide-4-(trifluoromethoxy)phenylhydrazone], and 1 mM each of rotenone and antimycin A was injected over 100 min to measure the respiration at different stages. Assay was normalized with total protein and analysis was done with XFe 2.0.0 software (Seahorse Biosciences).

### Immunoprecipitation

Cells were harvested in lysis buffer (50 mM Tris, 100 mM NaCl, 0.2 mM EDTA, 0.2 mM EGTA, 1% Triton X-100 with protease and phosphatase inhibitor cocktail). 40 μl of Protein A magnetic beads along with 500 μg of cell lysate and immunoprecipitation compatible primary antibody (1:50 dilution, CST) were incubated overnight at 4 °C in an orbital shaker. Immune complexes were separated by using magnetic GrIP RAC followed by three washes with lysis buffer and eluted proteins were analyzed by immunoblotting.

### Subcellular fractionation

RAW264.7 cells were harvested, centrifuged at 100 × *g* at 4 °C to pellet down and resuspended in Buffer 1 [150 mM NaCl, 50 mM HEPES pH 7.4, 25 µg/ml digitonin, protease inhibitor] followed by centrifugation to isolate cytosolic fraction in supernatant. Pellet was resuspended in Buffer 2 [150 mM NaCl, 50 mM HEPES, pH 7.4, 1%NP40, protease inhibitor] and incubated for 30 min followed by centrifugation at 7000 × *g* to pellet down and supernatant consisting of mitochondrial fraction. The pellet was resuspended in Buffer 3 [150 mM NaCl, 50 mM HEPES, 0.1% SDS, pH 7.4 with protease and phosphatase inhibitor] and vortexed periodically for 30 min followed by incubation at 4 °C for 1 h then centrifuged at 7000 × *g* for 10 min. Supernatant was collected containing the nuclear fraction.

### Flow cytometry

To assess different populations of macrophages from isolated LMs, cells were incubated with Mouse Seroblock (F_C_ receptor blocker) for preventing false-positive immunofluorescent signaling. For staining cell surface markers (CD45, F4/80/CD11b), cells were incubated with the antibody at1:100 dilutions in FACS buffer (0.5% BSA in PBS) for 30 min at 4 °C in dark. For intracellular marker staining of CD206, surface markers were stained before fixation/permeabilization (BD Cytofix/Cytoperm Kit, BD Biosciences). BD LSRFortessa was used for the assay and analysis was done with FlowJo software (BD Bioscience). For setting gates, unstained samples were used.

### Immunoblotting

Total cellular and tissue protein was prepared with lysis buffer containing 50 mM Tris-HCl (pH 7.4), 100 mM NaCl, 1 mM EDTA, 1 mM EGTA, and 1% Triton X-100 with protease/phosphatase inhibitor cocktail (Calbiochem). Protein samples were separated by SDS-PAGE and transferred to Immobilon-P membranes (Millipore). For detection, membrane was blocked with 5% non-fat dry milk in TBST for 1 h, followed by incubation with the specific primary antibody at 4 °C overnight and with horseradish peroxidase-labeled secondary antibodies for 1 h at room temperature. Signals were detected by chemiluminescence with Clarity Max^TM^ Western ECL substrate (Bio-Rad Laboratories) and scanned with a ChemiDocMP System (Bio-Rad). Details of antibodies used in this study are provided in Supplementary Table 1.

### qPCR

Total cellular as well as liver tissue RNA was isolated using Trizol (Thermo Fisher Scientific), cDNA was prepared from 1 µg of RNA by using e iScript First-Strand Synthesis Kit (Bio-Rad Laboratories). Quantification of gene expression was done by using SYBR green (Bio-Rad Laboratories) in Light Cycler 96 Real-Time PCR (Roche Diagnostics, Basel, Switzerland). Gene expression was normalized with 18 S rRNA expression by the ΔΔC_t_ method. Primer sequences are provided in Supplementary Table 2.

### Small interfering RNA transfection

Transient knockdown assay was performed by transfecting RAW264.7 cells with 30 pmol and 50 pmol of small interfering RNAs (siRNAs) specific for the HDAC3 gene against the sequences 1# 5’-GGAGCUGCUUAAGUACCACCCUC-3’, 2# 5’-GUGGCUACACUGUCC GAAAUG-3’ and 3# 5’-GGCAGACCUCCUGACGUAUG-3’ (Eurogentec, Liege, Belgium) by Lipofectamine^TM^RNAiMAX transfection reagent (Thermo Fisher Scientific).

### Chromatin Immunoprecipitation sequencing (ChIP-seq)

4 × 10^6^ RAW264.7 cells were used for ChIP assay using SimpleChIP® Enzymatic Chromatin IP Kit (9003S, Cell Signalling Technology) according to manufacturer’s protocol. Briefly, fixation and crosslinking of cells were done by 1% formaldehyde followed by glycine treatment. For nuclei preparation and chromatin digestion, two separate buffers were used containing DTT and Micrococcal Nuclease was used followed by sonication to disrupt the nuclear membrane. ChIPs were done with NF-κB p65 antibody (8242S, Cell Signalling Technology, MA, USA) in 1X ChIP buffer with ChIP grade Protein G magnetic bead. Elusion was done by heating the samples at 65 °C for 30 min followed by reversal of crosslinking by 5 M NaCl and Proteinase K. After DNA purification PCR was performed to check the promoter recruitment of p65 in specific genes for visualization agarose gel. ChIP-seq experiments were performed in two independent experimental replicates. Sequencing reads were aligned to the mm39 genome using Bowtie2 and further sorted using sambamba [41, 42]. MACS2 2.2.6 was used for peak calling. For both the treatments, peak calling was performed against the control bams and *p*-value set at 0.001. Further peak calls were combined using IDR 2.0.3 [43]. The combined peaks were then annotated using HOMER (v4.11.1) into GO categories [44]. To find out the differential binding between the two experimental groups DESeq2 was used from the DiffBind R package. The MA plot was constructed using the same package. Finally, for GO enrichment the chipenrich R package was used. Also all statistical tests have been performed in R (4.0.2).

### RNA sequencing and analysis

Isolated cDNA samples in triplicate were used for mRNA sequencing. A modified NEBNext RNA Ultra II directional protocol was used to prepare the libraries. Prepared libraries were sequenced on Illumina HiSeqX to generate 2 × 150 bp reads/sample. The raw reads were filtered using Trimmomatic for quality scores and adapters. Filtered reads were aligned to the Mus musculus genome using splice aware aligners like HISAT2 to quantify reads mapped to each transcript. Total numbers of uniquely mapped reads were counted using feature counts. VST normalization was done by using Deseq2 package in R. Differential expression analysis was done using Limma on Phantasus. Genes with an adjusted *p*-value < 0.05 were considered for further downstream analysis.

### Statistical analysis

Data represented as mean ± SD and Graphpad Prism 8 software was used for statistical analysis and representation. Unpaired two-tailed *t*-test and ANOVA were used to calculate statistically significant differences between two groups and for more than two groups, respectively. The cut-off value of *P* ≤ 0.05 was considered significant. No statistical methods were used to predetermine the sample size and variance.

## Supplementary information

Supplemental information

Original Data File

Checklist

## Data Availability

Mouse transcriptomic and ChIPseq data is available at the GEO repository (Accession number: GSE219094).
