# Supplementary material for: Butyrate limits inflammatory macrophage niche in NASH
Source: Cell Death Dis. 2023 May 18;14(5):332. doi: 10.1038/s41419-023-05853-6 (PMC10195803; doi:10.1038/s41419-023-05853-6)
Supplement: Supplementary file 1 — Supplemental information [file 41419_2023_5853_MOESM1_ESM.docx]

Supplementary Information

**Butyrate limits inflammatory macrophage niche in NASH**

Ankita Sarkar^1@^, Priya Mitra^1^, Abhishake Lahiri^2^, Tanusree Das^1^, Jit Sarkar^3^, Sandip Paul^4^,

Partha Chakrabarti^1@*^

^1^Division of Cell Biology and Physiology, CSIR-Indian Institute of Chemical Biology, Kolkata, India

^2^ Division of Structural Biology & Bioinformatics, CSIR-Indian Institute of Chemical Biology, Kolkata, India

^3^ Elucidata, New Delhi, India

^4^JIS Institute of Advanced Studies & Research, Kolkata, India

^@^Academy of Scientific and Innovative Research (AcSIR), Ghaziabad-201002, India

**Supplementary Table 1. Antibodies used.**

| **S. No.** | **Antibody** | **Company** | **Cat. No.** |
| --- | --- | --- | --- |
| 1. | TNF-α | CST | 11948S |
| 2. | β-Actin | Sigma-Aldrich | A5316 |
| 3. | IKKα | CST | 11930 |
| 4. | IKKβ | CST | 8943 |
| 5. | Phospho-IKKα/β | CST | 2697 |
| 6. | Phospho- NF-κB p65 | CST | 3033 |
| 7. | IκBα | CST | 4814 |
| 8. | Phospho- IκBα | CST | 2859 |
| 9. | NF-κB p65 | CST | 8242 |
| 10. | Acetyl-NF-κB p65 | CST | 12629S |
| 11. | Arg1 | CST | 93668S |
| 12. | Histone H3 | CST | 4499S |
| 13. | Cleaved Caspase3 | CST | 9661S |
| 14. | Stat1 | CST | 14994S |
| 15. | Phospho-Stat1 | CST | 9167S |
| 16. | Stat2 | CST | 72604 |
| 17. | Phospho-Stat2 | CST | 88410 |
| 18. | Stat3 | CST | 4904P |
| 19. | Phospho-Stat3 | CST | 9145S |
| 20. | Alexa 647  Cleaved Caspase3 | CST | 9602S |
| 21. | Inos | CST | 13120S |
| 22. | FITC. F4/80 | Invitrogen | 11-4801-82 |
| 23. | VioBlue. CD45 | MiltenyiBiotec | 130-110-802 |
| 24. | APC. CD11b | Invitrogen | 17-0112-82 |
| 25. | Pacific Blue. CD206 | Bio-Rad | MCA2235PB |
| 26. | PE-Cyanine7. CD80 | Invitrogen | 25-0801-82 |
| 27. | Cleaved IL-1β | CST | 52718S |
| 28. | Anti-Mouse IgG | Invitrogen | 31430 |
| 29. | Anti-Rabbit IgG | Invitrogen | 31460 |

**Supplementary Table 2.Primers for quantitative real time PCR and ChIP**

| **Genes** | **Forward Primer (5’-3’)** | **Reverse Primer (5’-3’)** |
| --- | --- | --- |
| *Tnf* | AATGGCCTCCCTCTCATCAGTT | CCACTTGGTGGTTTGCTACGA |
| *ll1b* | GGAGAACCAAGCAACGAC | GTACCAGTTGGGGAACTCTG |
| *ll6* | CCTCTGGTCTTCTGGAGTA | TTAGCCACTCCTTCTGTG |
| *Chil3*[Ym1] | TAAGGATGGCTACACTGG | CCAGTCTTAGAAGGGTCAC |
| *Retnla*[FIZZ1] | CCACTGTAACGAAGACTCTC | TCCAGTCAACGAGTAAGC |
| *Arg1* | CCAATGGTCAGGTTGATTCC | CCCACCCAGTGATCTTGACT |
| *RN18S* | GTTGGTTTTCGGAACTGAGG | TCGTTTATGGTCGGAACTACG |
| *Hdac3* | AATGTGCCCTTACGAGATGG | GTAGCCACCACCTCCCAGTA |

| **Genes** | **Forward Primer (5’-3’)** | **Reverse Primer (5’-3’)** |
| --- | --- | --- |
| *Tnf*promoter | CGGGGAGTCATACGGATTGG | TGAGTTTTCCACGGAGCCTC |
| *ll1b* promoter | TTCTGGGTGTGCATCTACGT | GTCATCGTGGTGGAAATGGG |
| *ll6* promoter | AGCACACTTTCCCCTTCCTA | ATCTTTGTTGGAGGGTGGGG |


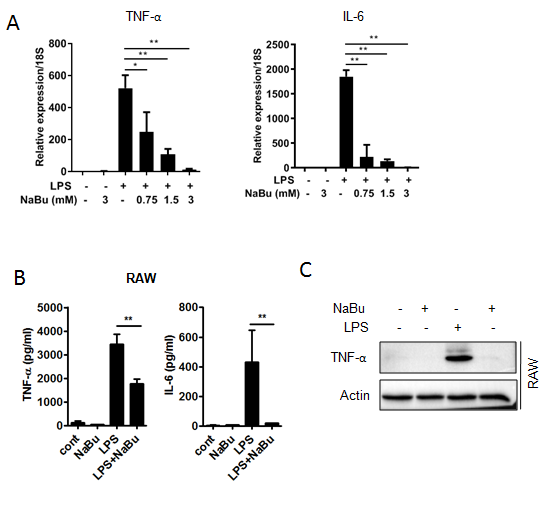


**Supplementary Figure 1. NaBu suppressed LPS induced RAW264.7cell activation**

RAW264.7 cells were pretreated with NaBu (doses 0.75, 1.5 and 3 mM) for 30 min followed by LPS (1μg/ml) stimulation for 4 h.(A) mRNA expression of TNF-α and IL-6 relative to 18S was analyzed.(B) Supernatants of aforementioned treatment conditions were analyzed for the presence of TNF-α and IL-6 by ELISA. (C) Expression of TNF-α was assessed by immunoblotting.Values were presented as mean ± SD, *P < 0.05; **P < 0.01.


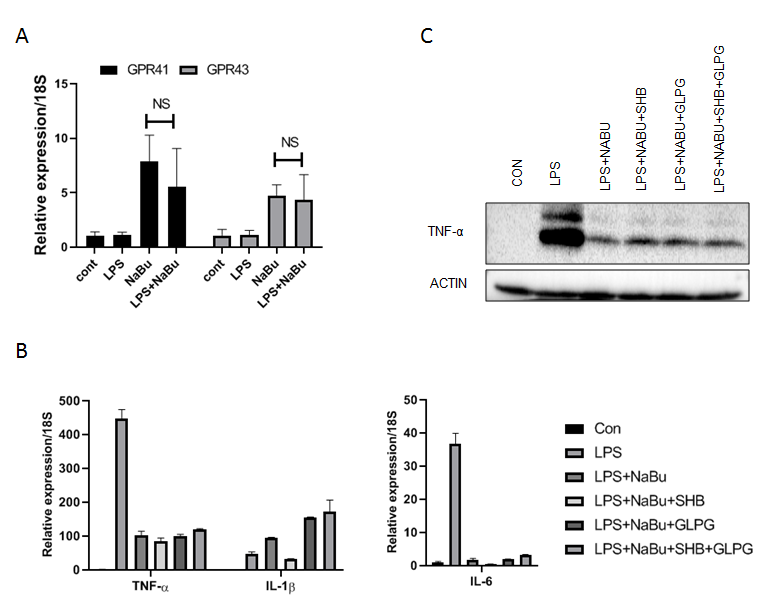


**Supplementary Figure 2. Anti-inflammatory function of NaBu is Independent of GPR41 and GPR43.**

(A) Gene expression of GPR41 and GPR43 relative to 18S in NaBu pre-treated RAW264.7 cells followed by 4 h of LPS (1µg/ml) stimulation.(B-C) RAW264.7 cells were pretreated withSodium 3-hydroxybutyrate (SHB) 5 mM [GPR41 antagonist] and GLPG0974 4 μM [GPR43 antagonist] individually and in combination followed by30 min NaBu (3 mM)and 4 h LPS (1µg/ml) stimulation (B) Gene expression analysis ofTNF-α, IL-1β, and IL-6, normalized to 18S expression (C) Immunoblot analysis of TNF-α.


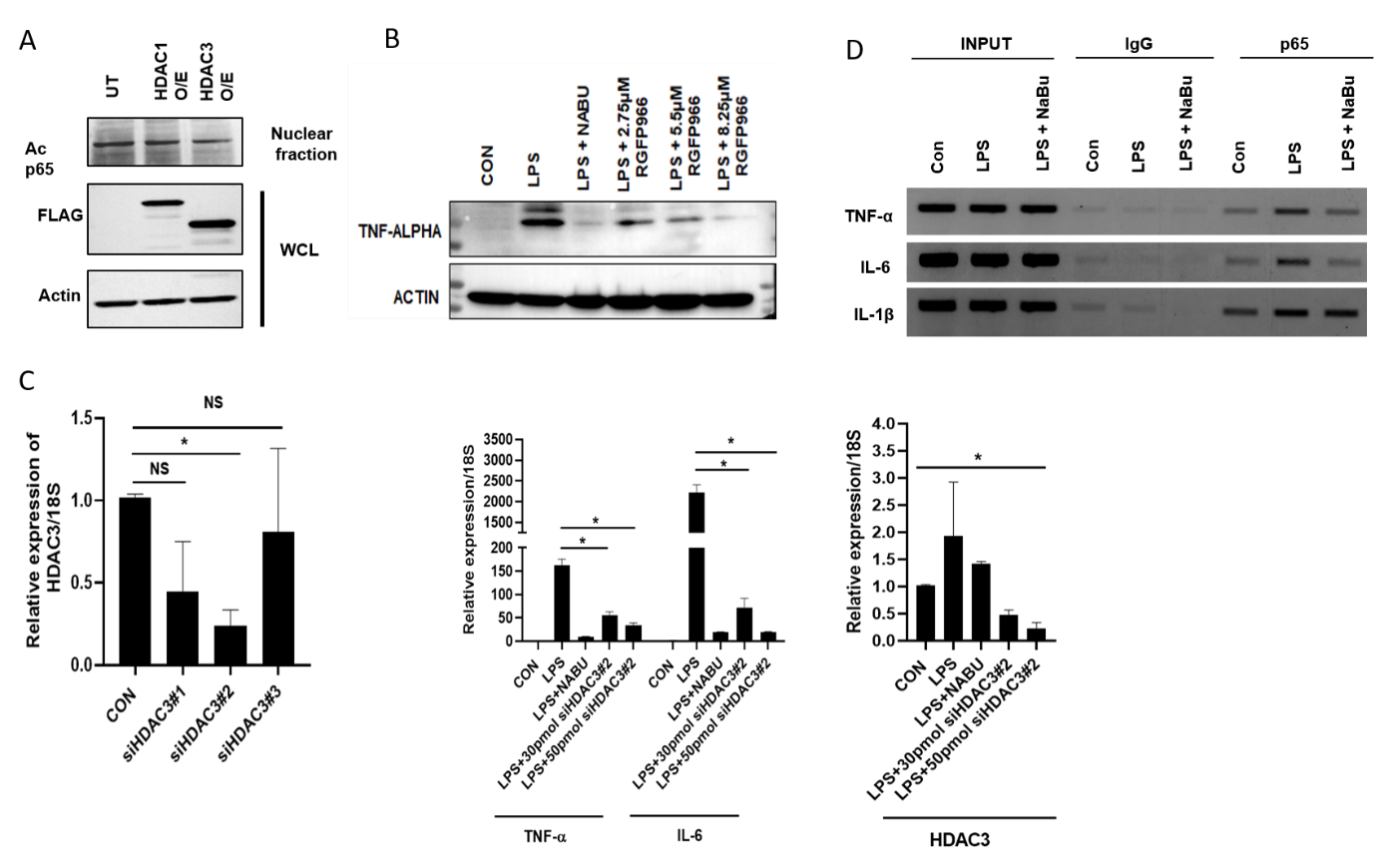


**Supplementary Figure 3. HDAC dependent p65 acetylation and chromatin recruitment.**

(A) HDAC1 and HDAC3 were overexpressed in HEK293A cells followed by subcellular fractionation and acetylated p65 levels were assessed by immunoblotting in nuclear fraction. (B) Immunoblot analysis of TNF-α in NaBu pretreated RAW264.7 cells followed by 4 h of LPS (1µg/ml) stimulation in the presence of RGFP966. (C) siRNA mediated HDAC3 knockdown using three different sequences. Pro-inflammatory gene (TNF-α, IL-6) expression analysis in the background of HDAC3 knockdown (sequence #2) followed by LPS (1µg/ml) activated RAW264.7 cells. (D) p65 recruitment to the TNF-α, IL-6 and IL-1β promoter was assessed by ChIP.


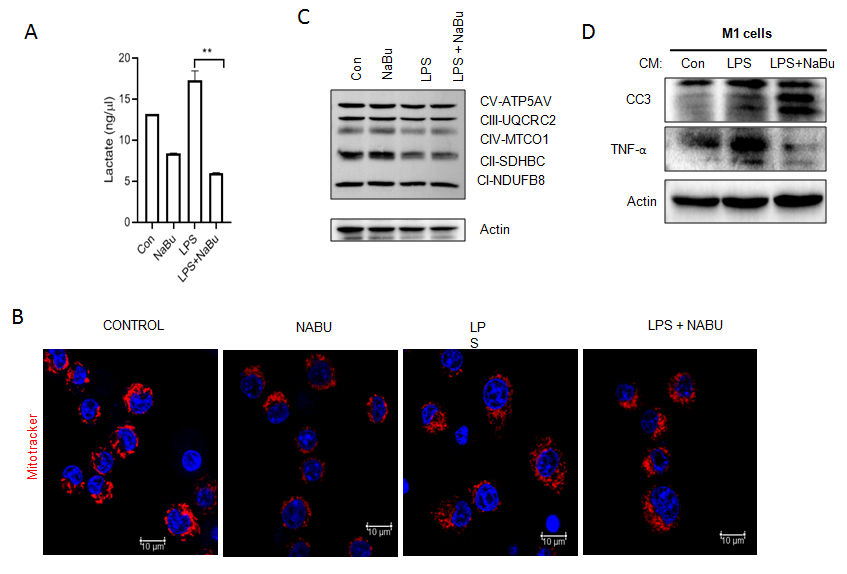


**Supplementary Figure 4. NaBu mediated metabolic regulation in RAW264.7 cells**

(A) Lactate levels were determined in NaBu (3 mM) pre-treated RAW264.7 cells followed by 4 h of LPS (1µg/ml) stimulation. (B)NaBu (3 mM) pre-treated RAW264.7 cells followed by 4 h of LPS (1 µg/ml) stimulation,incubated with 100 nM membrane potential–dependent MitoTracker Red CMX ROS for 30 min. Accumulation of MitoTracker Red was analyzed by f Confocal microscope. Hoechst (5µg/ml) was used to stain nuclei. (C) Expression of respiratory complex proteins. (D)Expression of CC3 and TNF-α by immunoblotting in RAW264.7 stimulated with LPS (1μg/ml) and IFN-ɣ (50ng/ml) for 16h followed by treated with conditioned media of control, LPS and LPS+NaBu treated RAW264.7 cells for 10 h.Values were presented as mean ± SD, **P < 0.01.
