# Supplementary material for: Butyrate limits inflammatory macrophage niche in NASH
Source: Cell Death Dis. 2023 May 18;14(5):332. doi: 10.1038/s41419-023-05853-6 (PMC10195803; doi:10.1038/s41419-023-05853-6)
Supplement: Supplementary file 2 — Original Data File [file 41419_2023_5853_MOESM2_ESM.pdf]

Full and uncropped western blot for Figure 1B

TNF-α

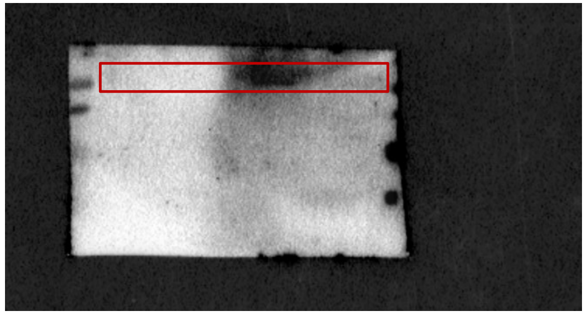

Actin

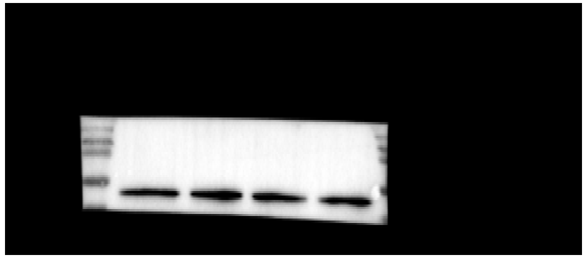

Full and uncropped western blot for Figure 1D

C. IL-1β

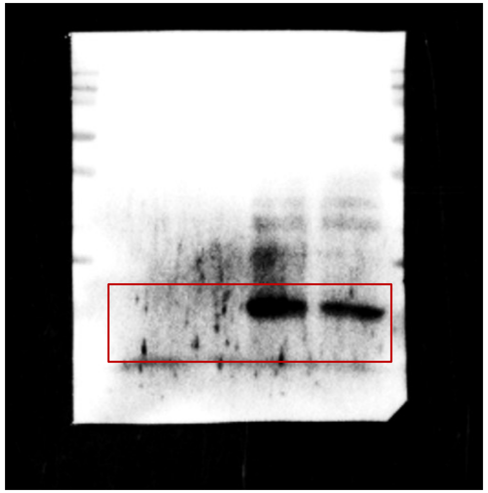

Full and uncropped western blot for Figure 1F

TNF-α

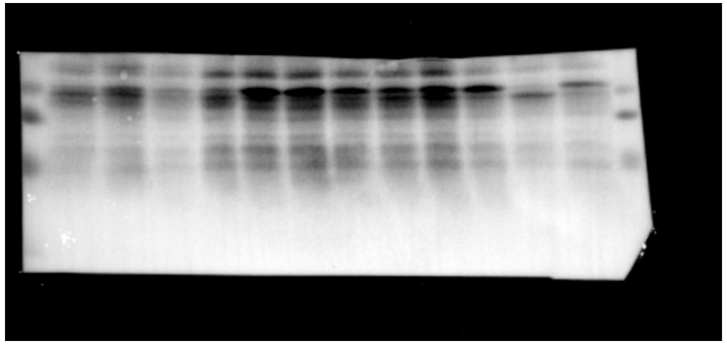

Actin

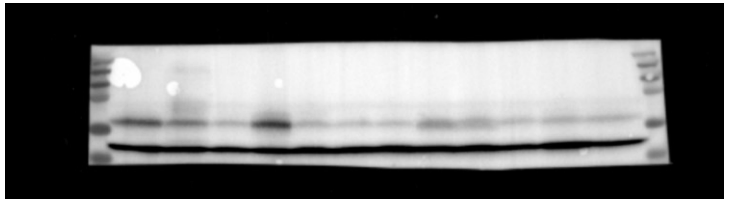

Full and uncropped western blot for Figure 2C

TNF-α

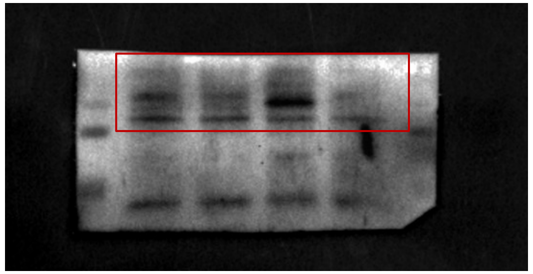

Actin

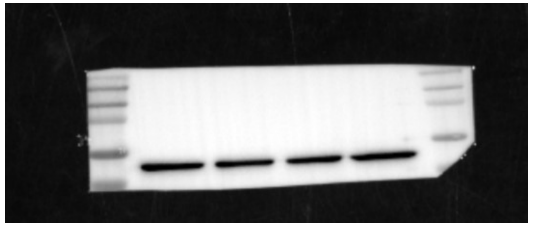

Full and uncropped western blot for Figure 2E

TNF-α

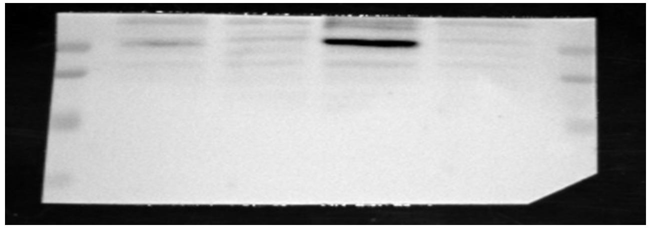

Actin

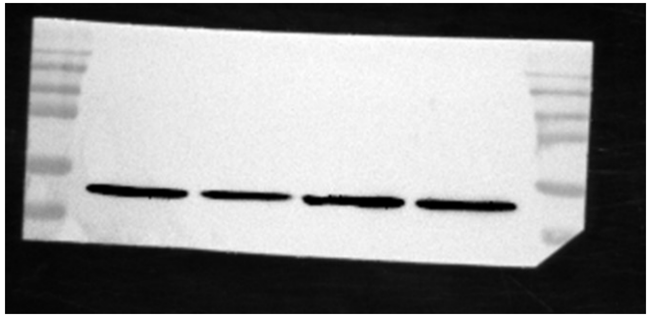

Full and uncropped western blot for Figure 2G

Arg1

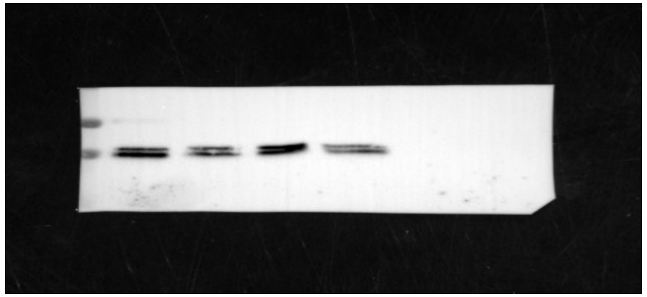

Actin

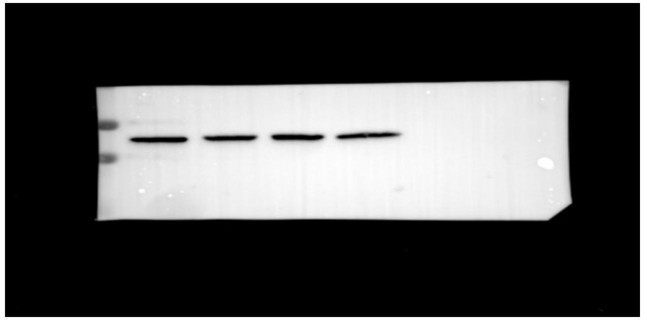

Full and uncropped western blot for Figure 3A

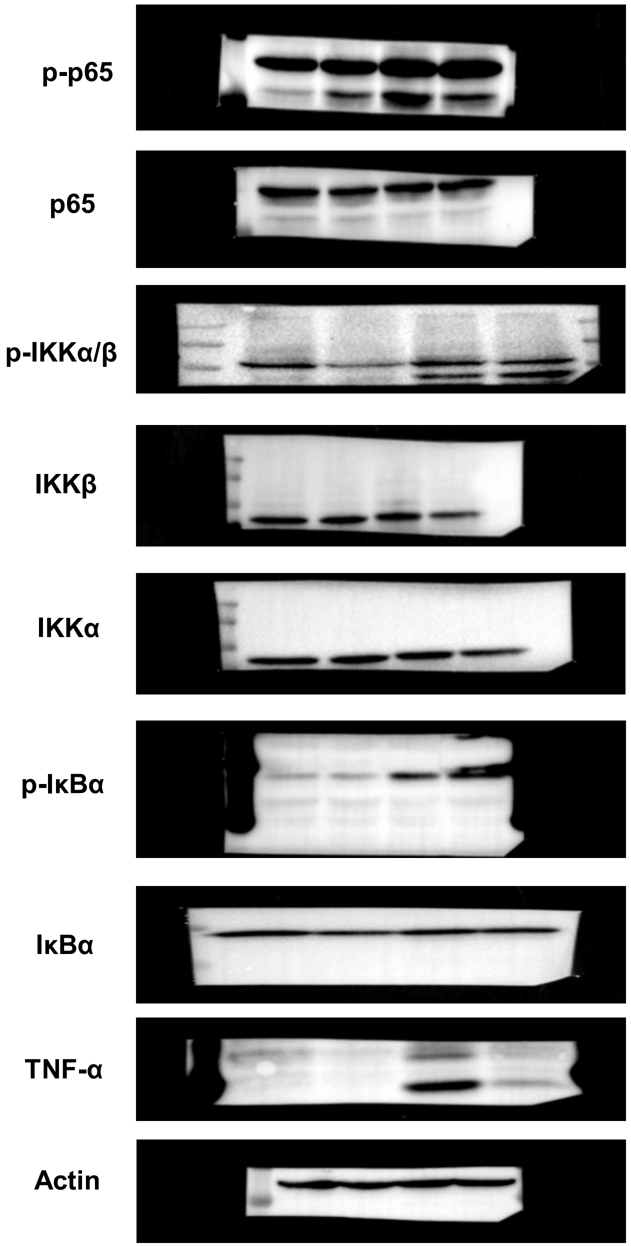

Full and uncropped western blot for Figure 3C

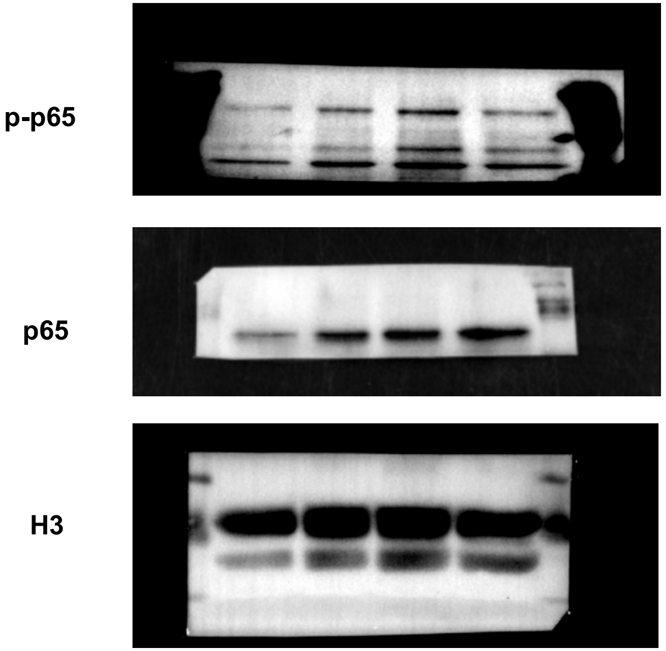

Full and uncropped western blot for Figure 3D

Ac p65

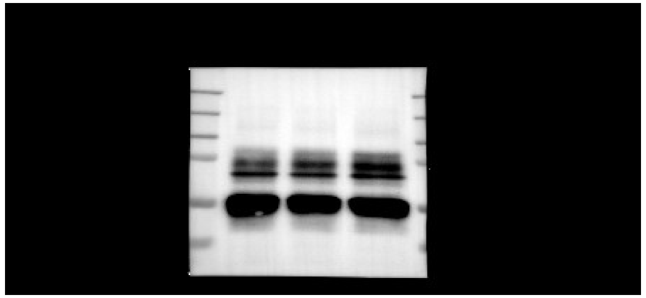

p65

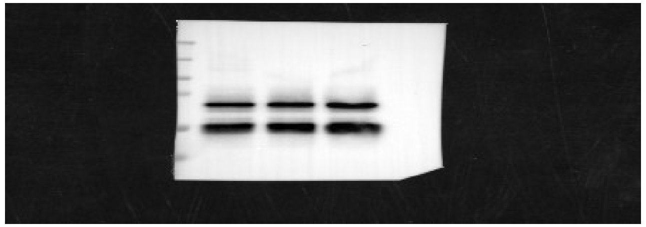

p65

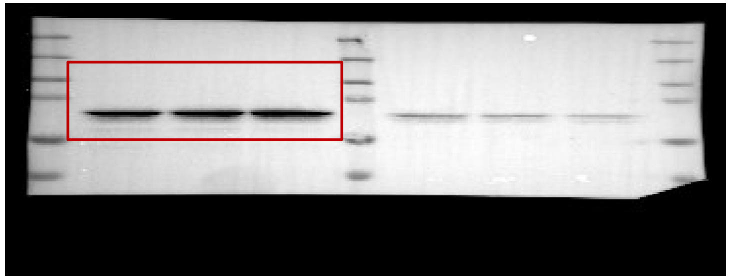

Full and uncropped western blot for Figure 3E

Ac p65

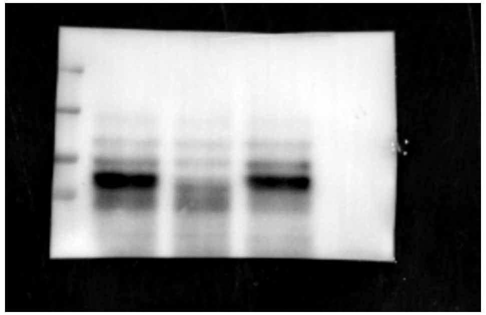

p65

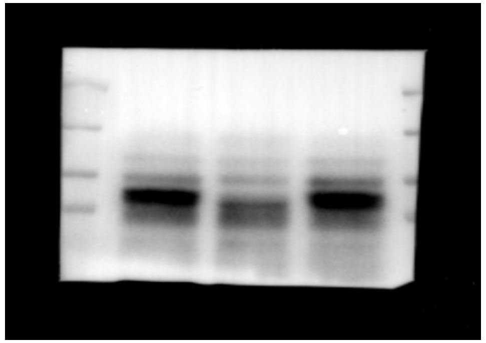

p65

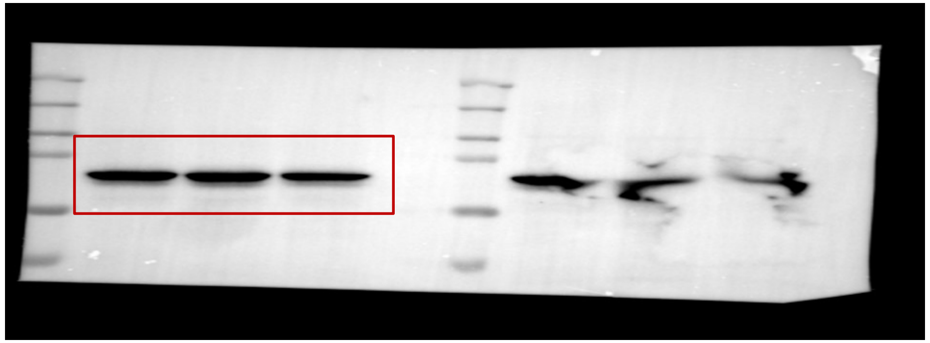

Full and uncropped western blot for Figure 3F

Ac p65

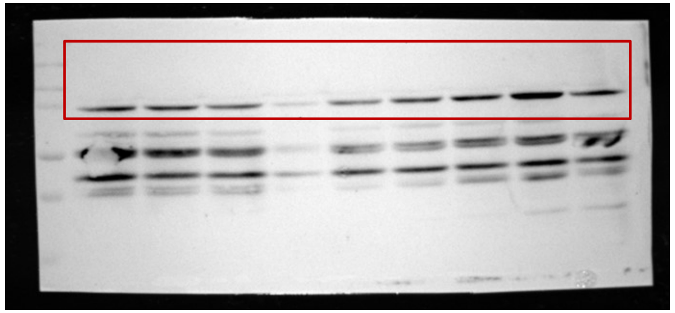

p65

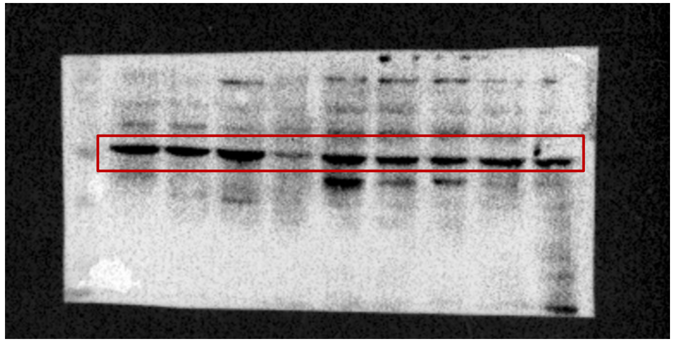

Actin

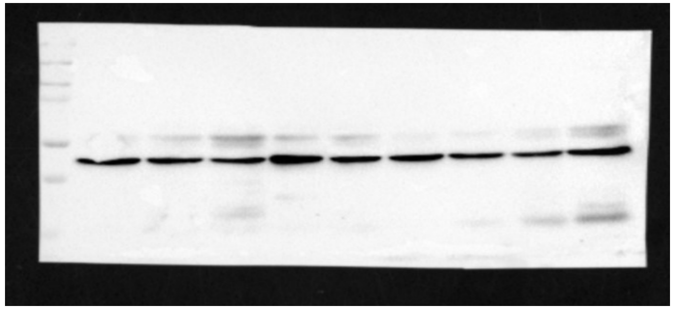

Full and uncropped western blot for Figure 4G

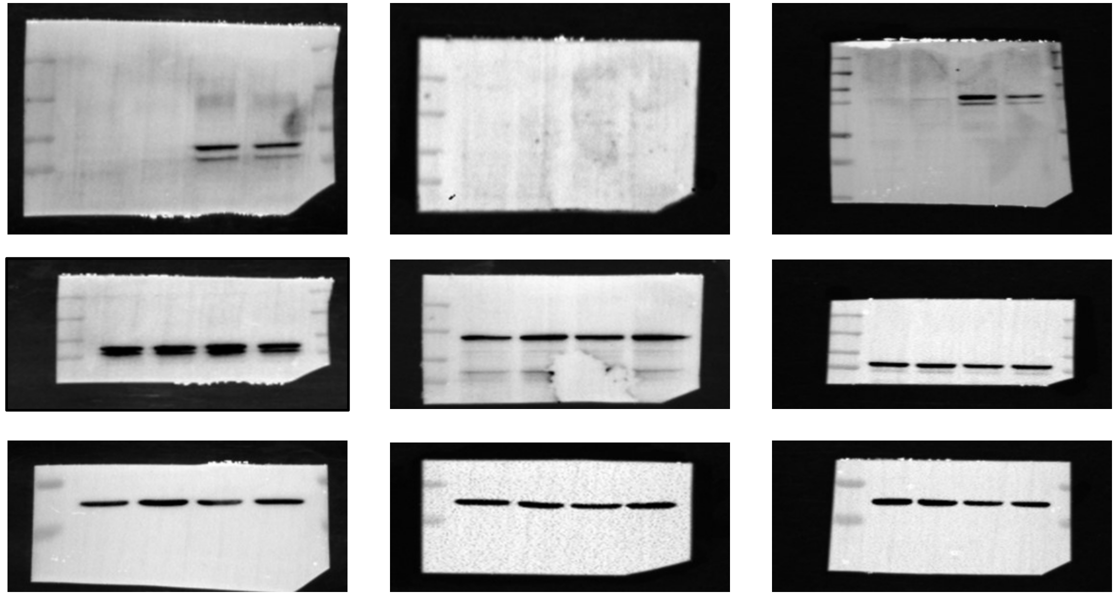

Full and uncropped western blot for Figure 5C

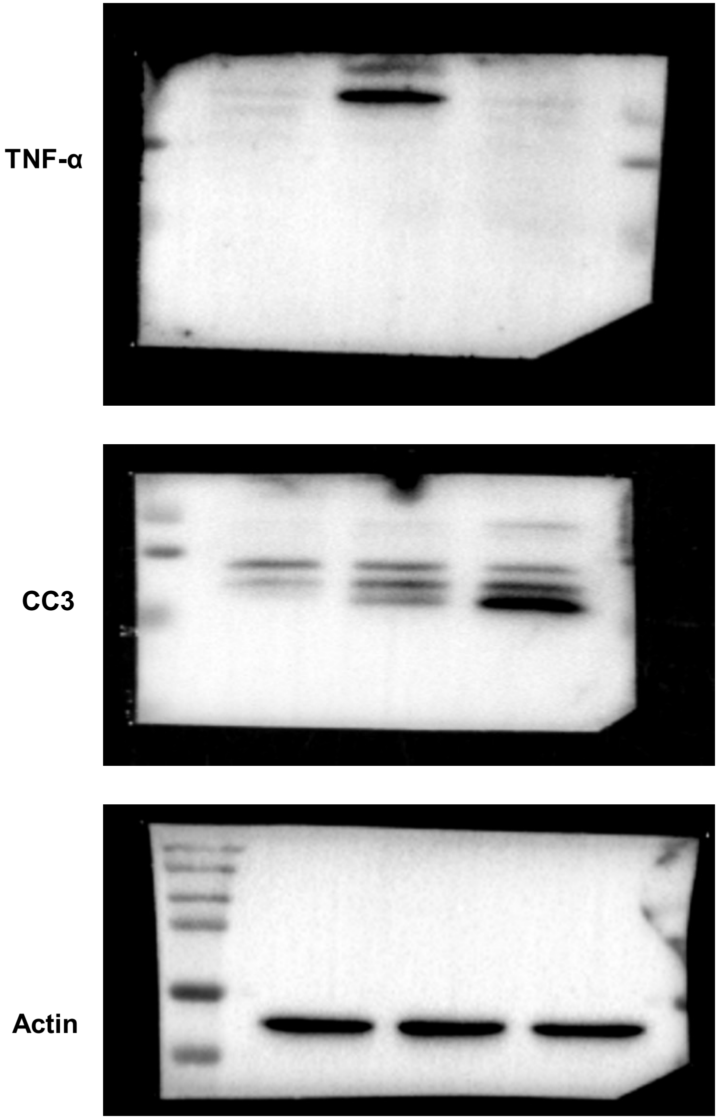

Full and uncropped western blot for Figure 5E

CC3

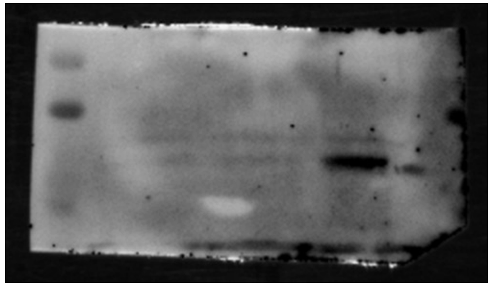

Actin

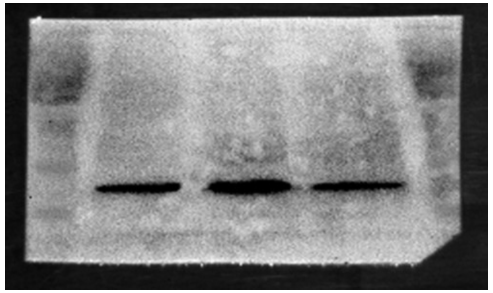

Full and uncropped western blot for Figure 6H

CC3

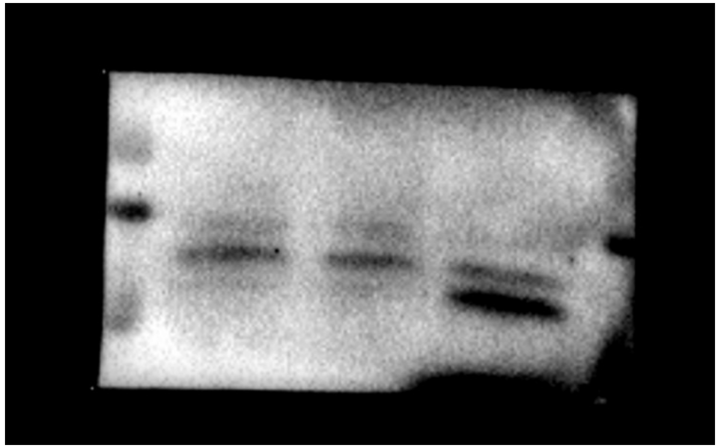

Actin

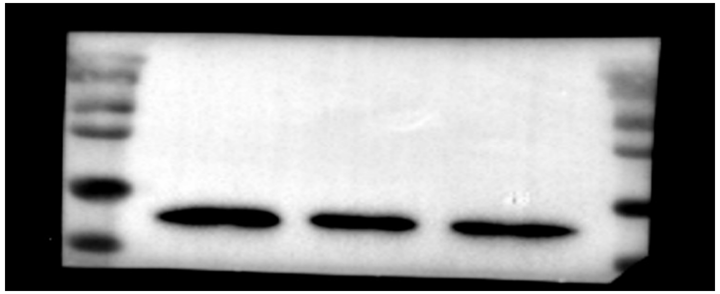

Full and uncropped western blot for Supplementary Figure 1C

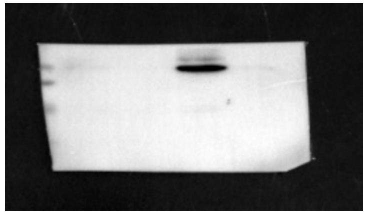

TNF-α

Actin

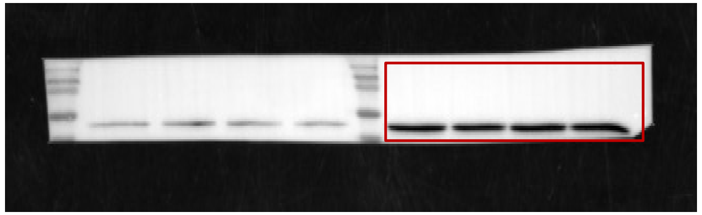

Full and uncropped western blot for Supplementary Figure 2C

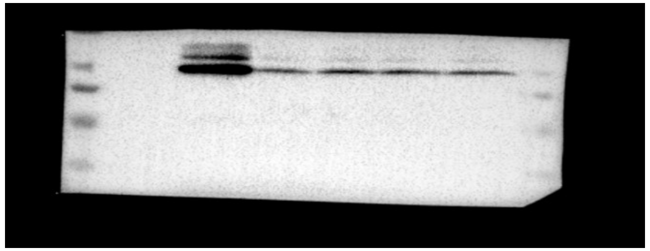

TNF-α

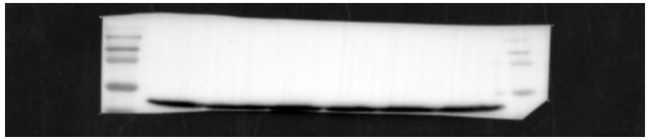

Actin

Full and uncropped western blot for Supplementary Figure 3A

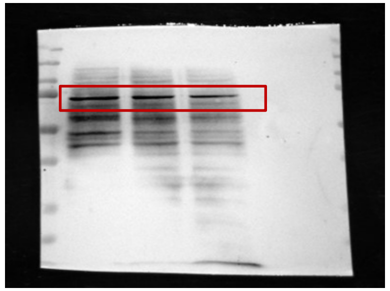

Ac p65

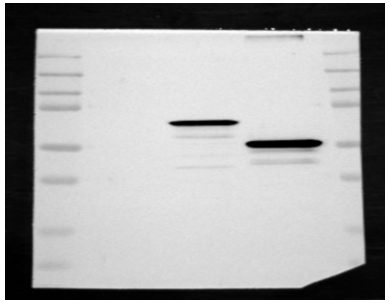

FLAG

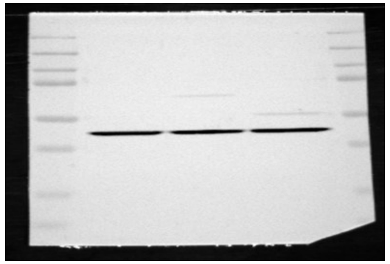

Actin

Full and uncropped western blot for Supplementary Figure 3B

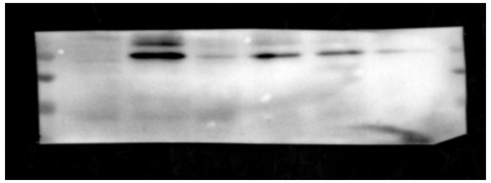

TNF-α

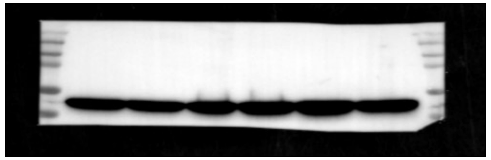

Actin

Full and uncropped western blot for Supplementary Figure 3C

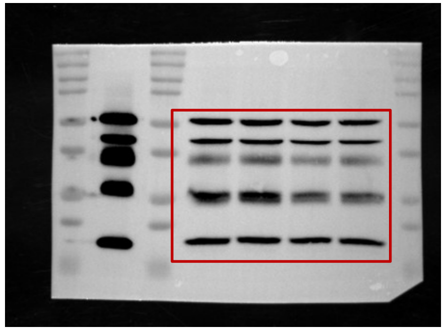

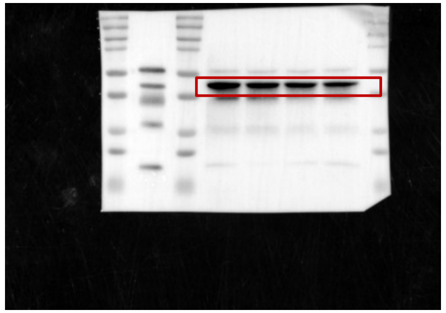

Full and uncropped western blot for Supplementary Figure 4D

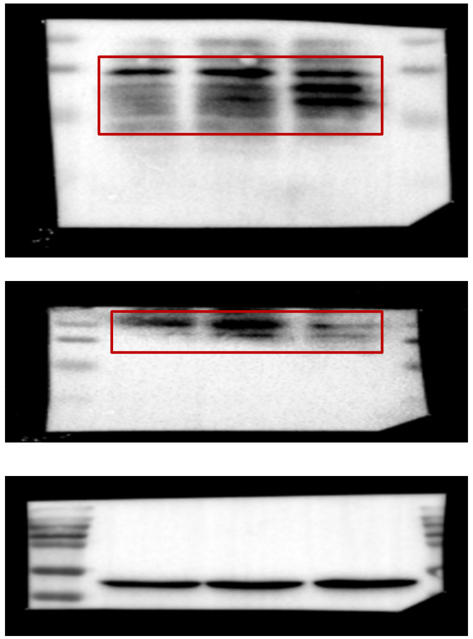

CC3

TNF-α

Actin
